# Genome-Wide Identification of 13 miR5200 Loci in Wheat and Investigation of Their Regulatory Roles Under Stress

**DOI:** 10.3390/genes16111349

**Published:** 2025-11-09

**Authors:** Yuan Zhou, Chenyu Zhao, Huiyuan Yan, Jiahao Yang, Mingyang Chen, Xia Wang, Pingfan Xie, Yongjing Ni, Jishan Niu, Jiangping Ren, Guojun Xia, Yongchun Li, Lei Li

**Affiliations:** 1 Henan Technology Innovation Centre of Wheat/National Engineering Research Centre for Wheat, Henan Agricultural University, Zhengzhou 450046, China; zy200102242023@163.com (Y.Z.);; 2 Shangqiu Academy of Agricultural and Forestry Sciences, Shangqiu 476000, China; 3 Henan Province Engineering Technology Research Center of High Resistance to Powdery Mildew Wheat, Zhengzhou 450046, China; 4 State Key Laboratory of High-Efficiency Production of Wheat-Maize Double Cropping, College of Agronomy, Henan Agricultural University, Zhengzhou 450046, China

**Keywords:** miR5200, bioinformatics validation, gene regulation, wheat stress response abiotic stress

## Abstract

Background/Objectives: miR5200 is miRNA unique to Poaceae plants. Induced under short-day conditions, it modulates flowering time by regulating the florigen *FT* gene expression. However, to date, the genetic locus responsible for mature miR5200 formation remains experimentally unvalidated, and its biological function in abiotic stress responses remains unknown. This has hindered systematic elucidation of miR5200’s physiological role and molecular mechanisms. Methods: This study utilized wheat as the research material. First, through bioinformatics analysis at the genomic level, 13 potential candidate tae-miR5200 gene loci were screened. Subsequently, the authenticity of these gene loci was systematically validated by combining tobacco transient transfection-based GUS staining assay and quantitative real-time PCR (qRT-PCR) to detect expression levels. Building upon this foundation, the expression patterns of tae-miR5200 under abiotic stresses such as low temperature, drought, and salinity, as well as SA, ABA, IAA, GA_3_, and MeJA treatments, were further investigated. Results: Experimental validation confirmed that 7 out of 13 potential gene loci are authentic and functional, and tae-miR5200 exhibited specific expression changes under different types of abiotic stress. Conclusions: This study confirms the authenticity of tae-miR5200 gene loci, effectively eliminating interference from bioinformatics-predicted false-positive loci in subsequent functional studies. It provides an experimental foundation for further investigation into the molecular mechanisms of tae-miR5200 in wheat responses to abiotic stress.

## 1. Introduction

Plants frequently encounter various abiotic stresses during natural growth, including low temperatures, drought, and saline–alkali conditions. These stresses severely impact growth processes, ultimately affecting yield and quality [1,2,3]. To counteract these adverse conditions, plants have evolved complex and sophisticated stress regulation mechanisms over long evolutionary periods, with post-transcriptional regulation playing a central role [4,5]. MicroRNAs (miRNAs), a class of non-coding small RNAs (sRNAs) approximately 22 nt in length [6], precisely regulate gene expression at the post-transcriptional level by specifically binding to target mRNAs [7,8,9]. Consequently, miRNAs play a crucial role in various physiological processes, including plant growth and development, organogenesis, and stress responses [10,11,12,13,14].

As a miRNA unique to Poaceae, the function of miR5200 has been validated in *Brachypodium distachyon*: its expression is induced by short-day conditions and influences flowering time by regulating the expression of the florigen *FT* gene [15]. However, current research on miR5200 has primarily focused on photoperiod regulation. Systematic research on its genomic localization and functional verification in wheat is still lacking, while its roles in other physiological processes such as stress responses and hormone regulation remain poorly understood. The realization of miRNA function primarily depends on the accurate generation of mature molecules [16].

The maturation of plant miRNAs is a multi-step, multi-factor, finely regulated process. First, microRNA genes are transcribed by RNA polymerase II into primary transcripts (pri-miRNAs) featuring hairpin structures [16]. Subsequently, the nuclear microprocessor complex (DCL1-HYL1-SERRATE) performs two sequential and precise cleavage steps: the first cleavage converts pri-miRNAs into precursor miRNAs (pre-miRNAs), and the second generates 21–22 nt miRNA-miRNA* duplexes [17,18,19,20]. Finally, HEN1-mediated methylation and AGO protein loading form functional miRISCs [21,22,23,24]. Dysfunction at any stage of this process may prevent mature miRNA production at the miRNA gene locus [25,26,27,28,29]. Structural integrity of pri-miRNA is a core prerequisite for miRNA biosynthesis; structural abnormalities directly cause processing initiation failure or processing aberrations [30,31,32]. This issue is particularly pronounced in wheat, given its large and complex genome, rendering numerous predicted miRNA gene loci unsuitable for functional studies. Therefore, the authenticity of miRNA gene loci is a prerequisite for miRNA function and a key factor for subsequent mechanistic research.

Based on this, we selected wheat as the research subject, screened 13 potential tae-miR5200 gene loci through bioinformatics analysis, and analyzed and validated their authenticity via tobacco transient transfection-based GUS staining assays combined with expression level analysis. Furthermore, to investigate the stress response mechanism of tae-miR5200, we hypothesized that it exhibits differential activation levels under stress and contains specific cis-elements that mediate this response. Subsequently, we further analyzed the expression pattern of tae-miR5200 under abiotic stress conditions.

This study aims to address the issue of false-positive miRNA gene loci interfering with functional research in bioinformatics predictions. It provides experimental evidence for elucidating the molecular mechanisms of tae-miR5200 in abiotic stress responses, while also providing new insights into crop stress-resistant molecular breeding and identifying functionally valid sites with potential application value.

## 2. Materials and Methods

### 2.1. Plant Materials

The wheat variety used in the experiment was “Jing 841,” which was sown at the end of October 2024 at the Yuanyang Base of Henan Agricultural University (Yuanyang County, Henan Province, China). For tissue-specific expression analysis, samples were collected from different tissues at the following stages: roots and young leaves during the overwintering period; stems, flag leaves, leaf sheaths, and young spikes during the heading stage; and grains at the filling stage. Tissue samples from every 3 plants were mixed and collected into the same centrifuge tube as one biological replicate, with 3 biological replicates set for each stage.

For stress experiments, “Jing 841” seedlings were cultured in nutrient solution (composition shown in Text S1) in a constant-temperature illumination incubator throughout the period (16 h light/24 °C, 8 h dark/22 °C, 60% humidity). First, plump and uniform wheat seeds were selected, disinfected with 10% NaClO solution for 15 min, rinsed thoroughly, and placed in Petri dishes. After soaking in sterile distilled water for 12 h, 600 germinated seeds (with exposed radicles) were selected and neatly arranged in seedling trays, with the nutrient solution replaced every 2 days. When the seedlings grew to the two-leaf-one-heart stage after 3 weeks, they were evenly divided into 6 groups in separate seedling trays for hormone stress treatment. The culture methods for wheat seedlings under low-temperature, salt, and drought stresses were the same as described above.

*Nicotiana benthamiana* plants were cultured for 3–4 weeks in a constant-temperature light incubator (16 h light/23 °C, 8 h dark/22 °C, 60% relative humidity) for use in transient transfection.

### 2.2. Analysis of Sequence Characteristics at Gene Loci

We performed sequence alignment analysis of the mature sequence of tae-miR5200 using the EnsemblPlants [33] database (http://plants.ensembl.org/Triticum_aestivum/Info/Index (accessed on 23 December 2024)) to obtain the corresponding gene sequence at the target site. Subsequently, we employed the online RNAFold Web Server (http://rna.tbi.univie.ac.at/cgi-bin/RNAWebSuite/RNAfold.cgi (accessed on 24 June 2025)) to predict the secondary structure of the sequence at this locus.

### 2.3. Prediction of Target Genes

Using the psRNATarget database (http://www.zhaolab.org/psRNATarget/ (accessed on 13 November 2024)) with the Chinese Spring wheat IWGSC RefSeq v1.0 as the reference genome sequence, we predicted the target genes of tae-miR5200. Filter the predicted target genes of tae-miR5200 with a maximum of 2 mismatches, where the mismatch between guanine (G) and uracil (U) is considered as 0.5 mismatches, referring to the method of Geng et al. [34]. The predicted target genes were then functionally annotated using the KEGG, GO, and KOG databases.

### 2.4. Vector Construction

To validate whether the gene locus mapped by the tae-miR5200 sequence can produce mature tae-miR5200, this study constructed three types of vectors: an overexpression vector driven by the constitutive 35S promoter (p35S) expressing the tae-miR5200 gene locus sequence (collectively termed p35S::MIR5200), a fusion expression vector expressing the *VRN3* coding sequence (CDS) with the *GUS* reporter gene (p35S::VRN3-GUS), and a fusion expression vector (p35S::mVRN3-GUS) for the *VRN3* mutant gene (abbreviated as *mVRN3*) obtained via overlapping extension PCR [35], wherein *mVRN3* carries a synonymous mutation at the VRN3-miR5200 binding site and is no longer regulated by tae-miR5200. The specific operational steps are as follows:

Based on the above three types of vectors, corresponding specific primers were designed (Table S1). Specifically, based on the prediction results of the secondary structure of the binding site sequence, a gene fragment approximately 300 bp in length was selected as the precursor sequence for the gene site, and specific primers were designed; based on the CDS of *VRN3* from the Chinese Spring wheat genome reference sequence IWGSC RefSeq v2.1, specific primers for the *VRN3* gene were designed; and site-directed mutations were introduced into the binding site sequence of *VRN3* and tae-miR5200, followed by the design of synonymous mutation primers. Detailed procedures for vector construction are provided in Text S2.

### 2.5. Agrobacterium-Mediated Tobacco Transient Transfection

The specific procedures for Agrobacterium-mediated transient transformation of tobacco are provided in Text S3. For GUS staining, the regions injected solely with the empty pCambia3301 vector (EV) and p35S::VRN3-GUS served as negative controls. For qRT-PCR, the expression level of VRN3 in the mixed injection of EV and p35S::VRN3-GUS was used as the negative control. The abaxial side of leaves from 3–4 week-old *N. benthamiana* was injected with the Agrobacterium suspension via a sterile syringe. All treatments for GUS staining and qRT-PCR used three injected tobacco leaves, representing three biological replicates. Following this injection step, the tobacco plants were placed in a constant-temperature incubator and incubated in darkness for a period of 36–48 h. The leaves were then collected for subsequent experiments.

### 2.6. Histochemical Staining for GUS Activity

Tobacco leaf samples infiltrated with Agrobacterium were collected for GUS staining. The corresponding volume of GUS staining solution (Coolaber, Beijing, China) was prepared based on the sample quantity. The tissue samples were completely immersed in the staining solution in a sterile After vacuum infiltration for 15 min at 0.08–0.1 MPa using a vacuum pump, the samples were placed in a 37 °C incubator for staining in the dark. Blue patches typically appear on the tobacco leaves after 6 h of staining. To remove chlorophyll and enhance the visibility of blue patches, gradient decolorization was performed using 70% ethanol, 80% ethanol, and 90% ethanol sequentially. Subsequently, the 90% ethanol decolorizing solution was replaced every hour until the leaf background turned completely white and the GUS staining blue patches were clearly visible. The GUS staining intensity in different injected regions was analyzed using ImageJ 1.0 software, and the staining intensity was represented by gray value.

### 2.7. Expression Level Detection

Take wheat or tobacco tissue samples, with three biological replicates per treatment. The design of primers was carried out via Primer Premier 5.0 software (www.premierbiosoft.com/primerdesign/index.html (accessed on 7 January 2025)). All primer pairs amplification efficiencies are all within the range of 90–110%, with specific single peaks in their melt curve plots and a melting temperature (Tm) ranging from 75 °C to 85 °C, and detailed primer sequences are shown in Table S2. qRT-PCR was performed following the method previously described by Jiao [36], with appropriate optimizations. Detailed experimental procedures are provided in Text S4. *Actin* served as the housekeeping gene for wheat, while *L25* was used for *N. benthamiana*. Triplicate reactions were set up for all samples, and gene expression levels were determined using the 2^−ΔΔCt^ method.

### 2.8. Prediction of Promoter Cis-Acting Elements

*Cis*-acting elements in the 2000 bp region upstream in the promoter of tae-miR5200 were predicted using PlantCARE (http://bioinformatics.psb.ugent.be/webtools/plantcare/html/ (accessed on 16 September 2025)). The classification of *cis*-acting elements was based entirely on the official functional annotations and classification criteria of the PlantCARE database. After prediction, the results were visualized using TBtools-II and Adobe Illustrator.

### 2.9. Stress Treatment

Low-temperature stress treatment: Wheat seedlings at the two-leaf-one-bud stage (with consistent growth status) were placed in a 4 °C constant-temperature incubator for sterile distilled water culture. At 0 h, 1 h, 2 h, 6 h, 12 h, 24 h, and 48 h post-treatment, leaf tissues were sampled from seedlings with uniform growth. Leaf samples from every 3 plants were mixed and collected into the same centrifuge tube, which served as one biological replicate. Three biological replicates were set for each time point. All samples were quickly frozen in liquid nitrogen, and stored at −80 °C.

Drought stress, salt stress, and their control treatments: Wheat seedlings at the two-leaf-one-bud stage (with consistent growth status) were cultured in a 24 °C standard constant-temperature incubator, with roots immersed in 20% PEG-6000 solution (drought stress), 150 mmol/L NaCl solution (salt stress), and sterile distilled water (control), respectively. Sampling times and storage methods were identical to those of the low-temperature stress treatment.

Hormone stress and its control treatments: Exogenous hormones (100 μM salicylic acid (SA), 100 μM abscisic acid (ABA), 10 μM indole-3-acetic acid (IAA), 100 μM gibberellin A_3_ (GA_3_), 100 μM methyl jasmonate (MeJA)) and sterile distilled water (control) were uniformly sprayed on both the adaxial and abaxial surfaces of wheat leaves, respectively. Spraying was stopped when water droplets condensed on the leaves and were about to drip, with a spray volume of 50 mL per treatment. Leaf tissue samples were collected from seedlings with uniform growth at different time points, following the same sampling principle and preservation method as mentioned above.

### 2.10. Data Processing

SPSS 26.0 software was used for multiple comparisons (α = 0.05), and Adobe Illustrator software was used for plotting. The data in this paper are expressed as mean ± standard deviation.

## 3. Results

### 3.1. Sequence Site Gene Analysis Characteristics

**Figure 1:**
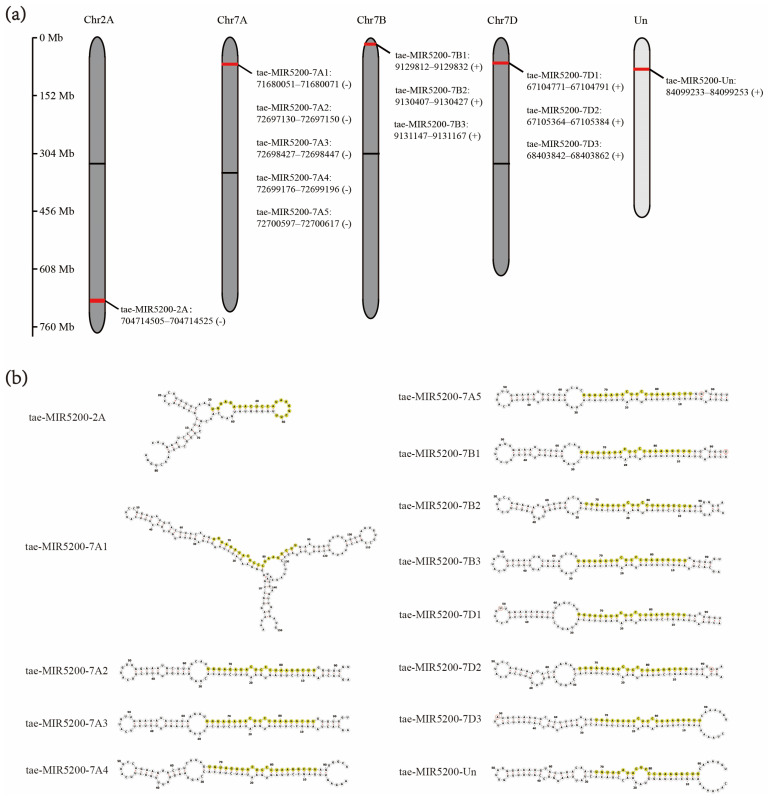
(**a**) Schematic diagram of the chromosomal locations of the 13 tae-miR5200 gene loci. (**b**) Schematic diagram of the secondary structure of the sequences at the 13 gene loci. Yellow indicates the mature sequence of tae-miR5200.

Homology alignment analysis of the tae-miR5200 sequence (retrieved from the miRBase database) against the Chinese Spring wheat genome reference sequence (IWGSC RefSeq v2.1) was performed via BLASTn. This analysis identified and named 13 tae-miR5200 gene loci (Figure 1a). Secondary structure prediction of these locus sequences revealed that all loci except tae-MIR5200-2A and tae-MIR5200-7A1 could fold into specific stem-loop structures (Figure 1b), with free energies below the threshold of −25 kcal/mol required for typical pre-miRNA stem-loop formation (Table 1).

### 3.2. Target Gene Prediction

**Figure 2:**
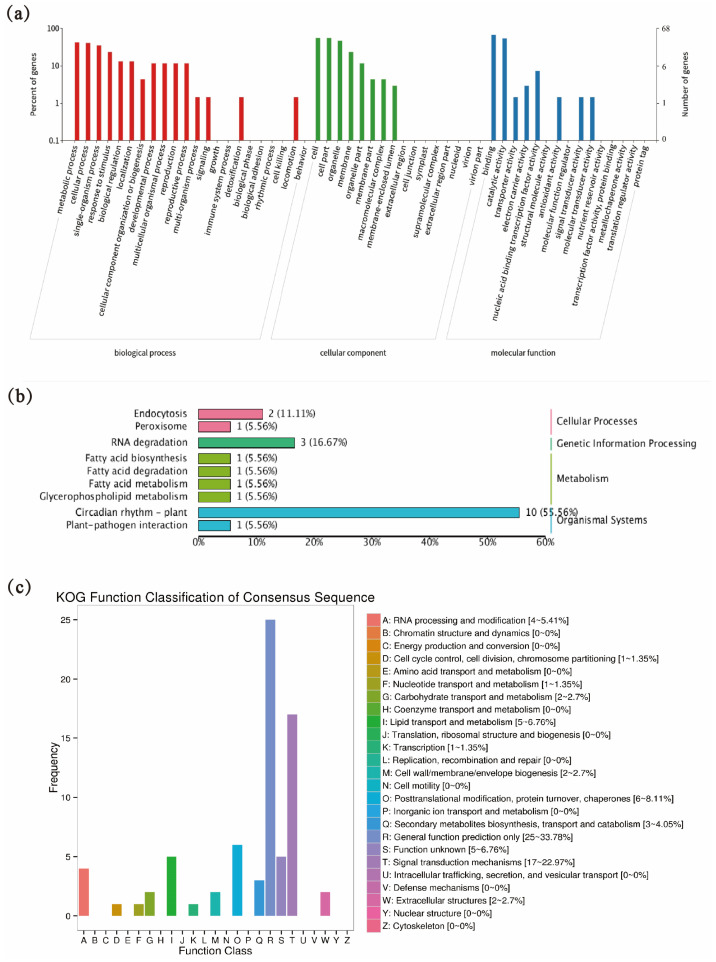
(**a**) Functional classification of target genes in the GO database. (**b**) Functional classification of target genes in the KEGG database. (**c**) KOG database analysis of target genes.

Subsequent gene locus identification requires target genes, so we predicted the target genes of tae-miR5200, identifying a total of 106 candidate target genes (Table 2 shows a subset of predicted target genes; Table S3 for the total of predicted target genes). These genes were functionally annotated in the KEGG, GO, and KOG databases (Figure 2). GO enrichment analysis revealed that tae-miR5200 target genes predominantly reside in organelles and cell membranes, participating in metabolic processes and responses to stimuli, and exerting effects through binding and catalytic activities (Figure 2a). KEGG functional annotation analysis revealed that nearly half of the tae-miR5200 target genes may be associated with plant circadian rhythms (Figure 2b). In the KOG database, target genes were primarily enriched in signal transduction mechanisms (Figure 2c). In summary, some target genes of tae-miR5200 are functionally enriched in stress-related regulatory networks such as response to stimuli and signal transduction, indicating that this microRNA may play a key role in wheat stress adaptation by regulating these target genes.

### 3.3. Target Gene Identification

**Figure 3:**
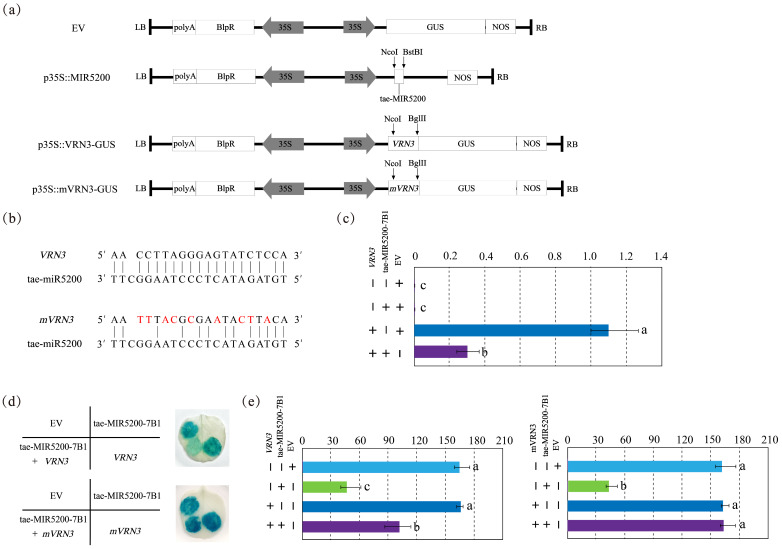
(**a**) Schematic diagram of vector structure related to the identification of tae-miR5200 target genes. (**b**) Base pairing characteristics between tae-miR5200 and its target gene *VRN3*. *mVRN3* is a mutant gene of *VRN3* carrying a synonymous mutation at the tae-miR5200 binding site, rendering its expression no longer regulated by tae-miR5200. Bases annotated in red indicate those with synonymous mutations. (**c**) *VRN3* expression analysis in different treatment samples from the tobacco transient transfection experiment. (**d**) Agrobacterium-mediated tobacco transient transfection GUS staining analysis. The left portion of the leaf shows the injection pattern schematic. (**e**) Analysis of staining intensity in different injected regions of tobacco transient transformation Gus staining assay. Analysis of variance (ANOVA) was used as the statistical method. Different lowercase letters indicate significant differences between treatments (*p* < 0.05). Error bars represent the standard error of the mean from three biological replicates.

According to miRBase annotations, the gene locus corresponding to the precursor sequence of tae-miR5200 is tae-MIR5200-7B1. Focusing on this locus, the present study selected the photoperiod gene *VRN3* as the key target from predicted candidates and constructed three vectors: an overexpression vector for the tae-miR5200 locus (p35S::MIR5200), a VRN3-GUS fusion expression vector (p35S::VRN3-GUS), and a *GUS* fusion vector with the *VRN3* synonymous mutant (*mVRN3*) (p35S::mVRN3-GUS) (Figure 3a,b). Target gene validation was conducted via tobacco transient transformation assays.

Results indicate that blue coloration appeared when injecting either the pCambia3301 empty vector (EV) or p35S::VRN3-GUS alone, confirming normal *GUS* gene expression with consistent intensity. Injection of the p35S::MIR5200 alone did not produce blue coloration. However, when p35S::MIR5200 was co-injected with the p35S::VRN3-GUS at equal volumes, *GUS* gene expression intensity was significantly weaker than that observed with p35S::VRN3-GUS injection alone, indicating that tae-miR5200 downregulates *VRN3* expression (Figure 3d,e). Following injection of p35S::mVRN3-GUS into tobacco leaves using the same method, the blue color intensity in areas where p35S::MIR5200 and p35S::mVRN3-GUS were co-injected matched that observed with p35S::mVRN3-GUS alone. This confirms that tae-miR5200 targets *VRN3*, with the binding site aligning with the mutated region (Figure 3d,e).

To further validate the authenticity of the results, we examined *VRN3* expression levels under different injection conditions. Findings revealed that when p35S::MIR5200 was co-injected with p35S::VRN3-GUS, *VRN3* expression was significantly lower than when p35S::mVRN3-GUS was injected alone. In contrast, co-injection of p35S::MIR5200 with p35S::mVRN3-GUS yielded *mVRN3* expression levels nearly identical to those observed with p35S::mVRN3-GUS alone. This further confirms that tae-miR5200 specifically targets *VRN3* and binds to the mutated region of *mVRN3* (Figure 3c).

### 3.4. Identification of Genetic Locations

**Figure 4:**
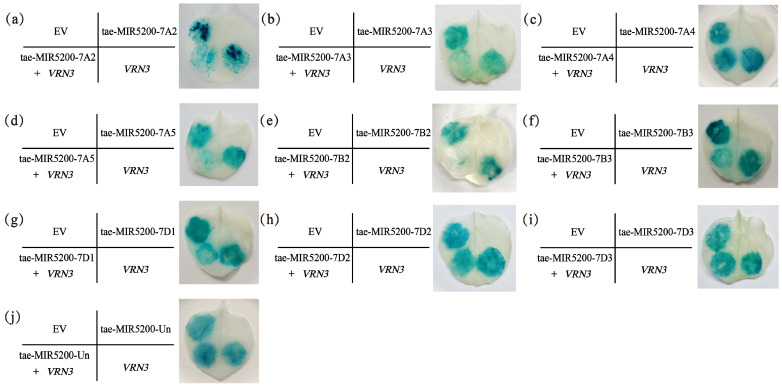
(**a**–**j**) Identification of 10 gene loci for tae-miR5200. Agrobacterium-mediated transient transfection in tobacco with GUS staining analysis. The left portion of the leaf shows the injection pattern schematic.

**Figure 5:**
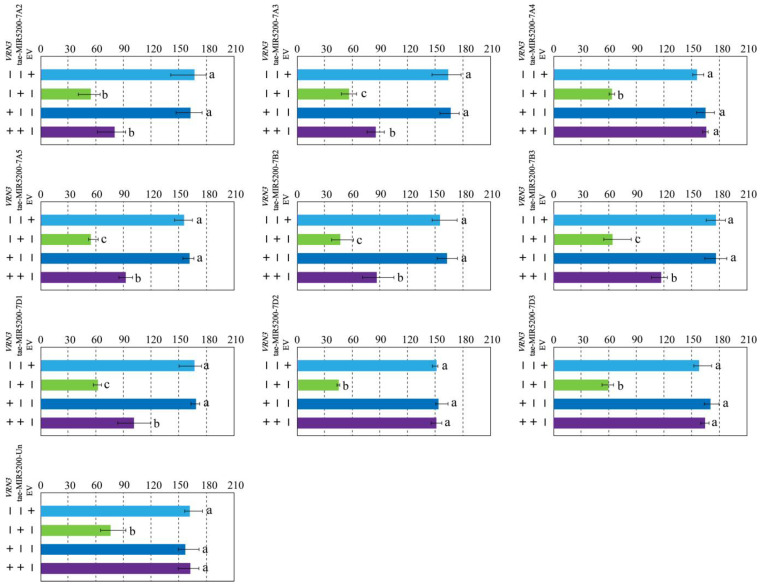
Identification of 10 gene loci for tae-miR5200. Analysis of staining intensity in different injected regions of tobacco transient transformation GUS staining assay. Analysis of variance (ANOVA) was used as the statistical method. Different lowercase letters indicate significant differences between treatments (*p* < 0.05). Error bars represent standard error of the mean from three biological replicates.

**Figure 6:**
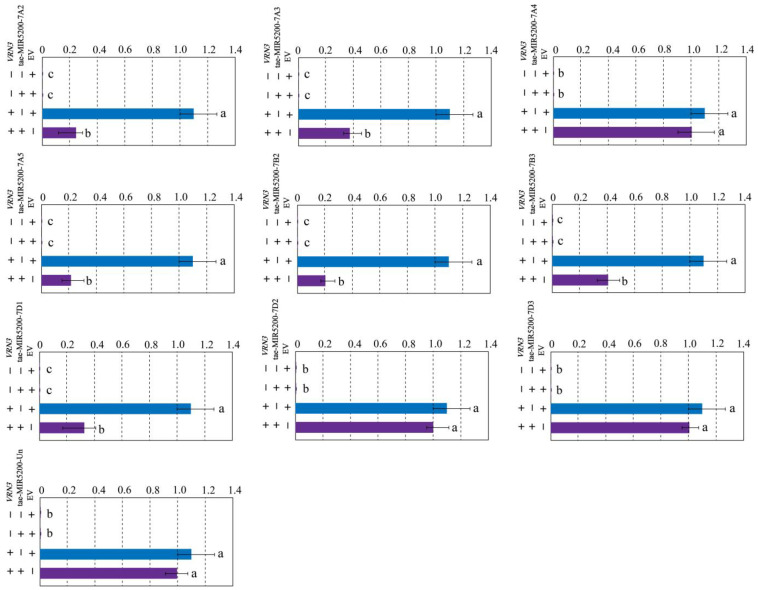
Identification of 10 gene loci for tae-miR5200. Analysis of *VRN3* expression in different treatment samples from tobacco transient transfection experiments. Analysis of variance (ANOVA) was used as the statistical method. Different lowercase letters indicate significant differences between treatments (*p* < 0.05). Error bars represent standard error of the mean from three biological replicates.

To validate whether the gene locus identified through sequence alignment of tae-miR5200 can produce mature miR5200, we utilized the negative regulatory effect of miR5200 on its target gene *VRN3* to identify the gene locus via tobacco transient transformation experiments. Results showed that both EV and p35S::VRN3-GUS injections alone produced blue coloration in the injected regions. Injections of the p35S::MIR5200 alone did not produce blue coloration. However, the blue color intensity in regions injected with p35S::MIR5200-7A2/7A3/7A5/7B2/7B3/7D1 co-injected with p35S::VRN3-GUS exhibited significantly weaker blue coloration than p35S::VRN3-GUS alone (Figure 4a,b,d–g and Figure 5). This confirms that all 6 gene loci (tae-miR5200-7A2/7A3/7A5/7B2/7B3/7D1) can produce mature tae-miR5200 and target *VRN3* for suppression. Simultaneously, we measured *VRN3* expression levels under different injection conditions. Results showed that when p35S::MIR5200 was co-injected with an equal amount of p35S::VRN3-GUS Agrobacterium strain, *VRN3* expression was significantly lower than when the p35S::VRN3-GUS Agrobacterium strain was injected alone, further confirming that tae-miR5200 specifically suppresses *VRN3* (Figure 6).

### 3.5. Promoter Element Analysis

**Figure 7:**
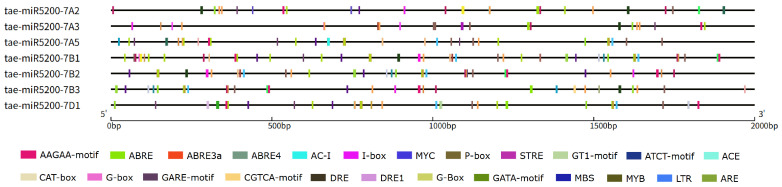
Schematic diagram of *cis*-acting elements in the promoter region of the tae-miR5200 gene loci.

To further elucidate the key factors regulating the transcription and maturation of tae-miR5200, we conducted *cis*-acting element analysis on the 2000 bp upstream region of the 6 gene loci generating mature miR5200. Results revealed that this region contains core promoter elements such as TATA-box and CAAT-box, confirming its promoter activity. In addition, multiple functional response elements were enriched, including light-responsive elements such as G-box, I-box, and Sp1; abiotic stress-responsive elements such as LTR, MBS, and ARE; and hormone-responsive elements such as ABRE and as-1. Among the seven tae-miR5200 gene loci, all contain G-box, ARE, and MYB elements, six harbor ABRE and MYC elements, and five possess MBS elements. The presence of these elements suggests that tae-miR5200 may play a crucial role in wheat growth and development as well as in responses to abiotic stresses by integrating multiple signals such as light, stress, and hormones (Figure 7, Table S4).

### 3.6. Analysis of Expression Patterns

**Figure 8:**
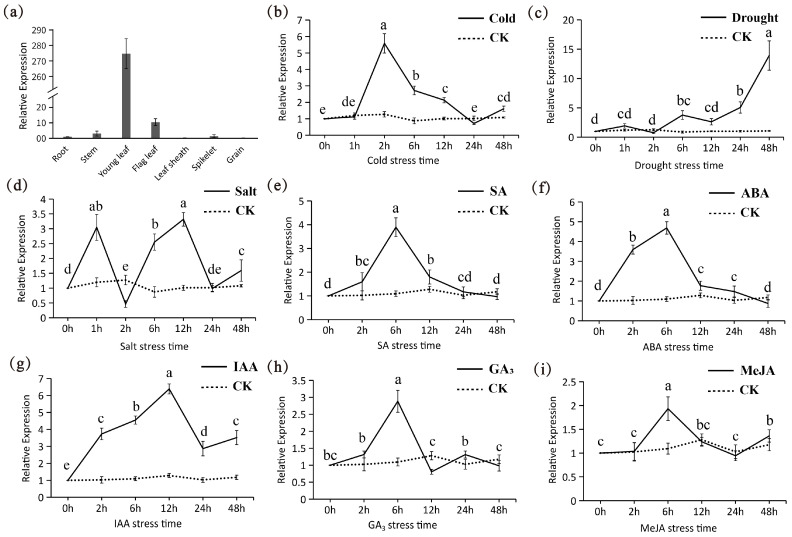
(**a**) Tissue expression analysis of tae-miR5200. (**b**–**i**) Expression pattern analysis of tae-miR5200 under different stress treatments. Analysis of variance (ANOVA) was used as the statistical method. Different lowercase letters indicate significant differences between treatments (*p* < 0.05). Error bars represent the standard error of the mean from three biological replicates.

Tissue expression analysis revealed that tae-miR5200 is exclusively expressed in wheat leaves, with significantly higher expression levels in young leaves compared to flag leaves. This indicates that young leaves may serve as the primary site where tae-miR5200 participates in wheat physiological regulation (Figure 8a). Based on these tissue expression patterns, to validate the functional significance of the stress response elements, we used wheat young leaves as experimental material and analyzed the expression dynamics of tae-miR5200 under different abiotic stress and hormone treatments via qRT-PCR.

Under low-temperature stress, the expression level of tae-miR5200 exhibited a “first-rise-then-decline” trend with prolonged treatment duration, reaching its peak expression at 2 h post-treatment (Figure 8b). Under drought stress, the gene expression level continuously increased with extended stress duration, reaching 14-fold the control level at 48 h (Figure 8c). Under salt stress, the expression response of tae-miR5200 exhibited significant fluctuations, showing marked upregulation at both the 1 h and 6 h time points. In hormone stress treatments, the expression of tae-miR5200 generally followed a common “upregulation followed by downregulation” pattern, but the response characteristics differed among various hormone treatments. Specifically, under SA, ABA, GA_3_, and MeJA treatments, tae-miR5200 reached its expression peak at 6 h post-treatment. In contrast, the expression peak for IAA treatment occurred at 12 h, followed by a gradual decline until a slight re-upregulation at 48 h. Similarly, MeJA treatment exhibited a slight re-upregulation at 48 h.

The results indicate that tae-miR5200 not only responds specifically to hormonal signals but also participates in the response processes to abiotic stresses such as low temperature, salinity, and drought. This finding not only provides experimental support for the functional validity of stress response elements identified in previous promoter analyses but also further confirms that tae-miR5200 is closely associated with wheat growth and development as well as adaptation to abiotic stress.

## 4. Discussion

The formation of mature miRNAs relies on the transcription of miRNA gene loci in the genome and a subsequent series of processing events [37,38,39]. Many potential loci may fail to be transcribed or processed into functionally active mature miRNAs due to genomic epigenetic modifications [39,40,41,42], insufficient transcription initiation efficiency [43,44,45,46], or defects in processing enzyme recognition [47,48]. Specifically, if the stem extension length or folding free energy of the secondary structure of the pri-miRNA hairpin deviates from physiological thresholds, the pri-miRNA cannot adopt the “functional conformation” required for DCL1 recognition, ultimately disrupting precise cleavage site localization [49]. Furthermore, high-frequency base mismatches or atypical bulge structures within the double-stranded RNA (dsRNA) region of the pri-miRNA stem [50,51,52,53,54], or the absence of the internal loop specifically recognized by the PAZ domain of DCL1 [55,56], disrupts the targeted binding and substrate stabilization efficiency between DCL1 and pri-miRNAs. This causes the processing pathway to stall at its initial stage, thereby impairing mature miRNA biogenesis.

During the comprehensive analysis and experimental validation of the 13 identified tae-miR5200 gene loci, six pseudogene loci were characterized. These loci were incapable of producing mature miR5200. Among these, the tae-MIR5200-2A and tae-MIR5200-7A1 pseudogene sites were unable to fold into the typical stem-loop structure essential for miRNA biosynthesis due to their inherent sequence characteristics. While tae-MIR5200-7A4 and tae-MIR5200-Un could form stem-loop structures, their lower stems exhibited branching, which directly prevented DCL1 from effectively binding to the pri-miRNA via its DUF283 module and PAZ domain, constituting the primary reason why these two loci failed to produce mature miR5200. Furthermore, while the secondary structure of tae-MIR5200-7D3 shows no significant differences from that of other functional gene loci, the abnormal expansion of the first small loop region in its lower stem is hypothesized to have interfered with DCL1 recognition and binding, ultimately inhibiting mature miR5200 production (Table 3).

From a functional perspective, this study confirms the evolutionary conservation and pleiotropy of tae-miR5200. Previous studies have shown that miR5200 in *B. distachyon* regulates the plant photoperiod process by targeting the key flowering gene *FT* [12]. In contrast, this study reveals that wheat tae-miR5200 specifically targets *VRN3*, a member of the *FT* family. As a crucial regulatory gene for wheat growth and development, *VRN3* encodes a RAF kinase inhibitor-like protein [57], which forms a protein complex with transcription factor FDL2 and 14-3-3 [58,59,60]. This complex activates the key flowering gene *VRN1* while suppressing the functions of negative flowering regulators *VRN2* and *ODDSOC2* [61,62,63]; these coordinated regulatory effects promote the transition of the shoot apical meristem from the vegetative to the reproductive phase, thereby facilitating flowering [64,65]. Thus, tae-miR5200 can directly intervene in wheat growth and development by targeting this critical hub (*VRN3*). This mechanism provides precise, controllable molecular targets and technical approaches for wheat breeding.

Functional association analysis of tae-miR5200 gene locus-derived *cis*-acting elements and its candidate target genes revealed that the promoter region of the tae-miR5200 gene locus is enriched with typical light-responsive *cis*-acting elements such as G-box and I-box, further corroborating its conserved function in the photoperiod and flowering regulation pathways. Previous studies identified miR5200 through small RNA sequencing analysis following drought stress treatment, suggesting that this miRNA may be involved in the response to abiotic stress [66]. Additionally, *cis*-acting elements for stress and hormone responses, including LTR, MBS, and ABRE, were identified. Combined with subsequent expression analyses under abiotic stress and hormone treatments, this suggests its own expression may be co-regulated by abiotic stress and hormone signals. Annotation of tae-miR5200 candidate target genes indicated that these genes are widely involved in abiotic stress response and cellular signal transduction pathways. These dual analyses confirm that the physiological function of tae-miR5200 extends beyond photoperiod regulation, potentially integrating hormonal and environmental signals to participate in wheat’s abiotic stress response processes.

This study still has certain limitations regarding the identification of the tae-miR5200 gene locus and the verification of its functional role in abiotic stress response. Specifically, the current identification of this gene locus relies solely on indirect evidence from GUS staining assays, and direct detection of the mature miRNA has not been performed using techniques such as Northern blot or Small RNA sequencing. Additionally, the inference of its involvement in stress regulation is only based on indirect clues from abiotic stress-responsive element analysis of the promoter, expression pattern analysis, and target gene function prediction, lacking verification of the actual regulatory effects through phenotypic observation and physiological index determination of wheat plants. For future research, we plan to construct tae-miR5200 overexpression and knockdown transgenic wheat lines, validate the gene locus identification results of this study via transcriptome sequencing, and verify the binding relationship between stress-related transcription factors and the tae-miR5200 promoter region using ChIP-PCR. Furthermore, combined with phenotypic observation and stress tolerance-related physiological index determination after abiotic stress treatment, the functional regulatory mechanism of tae-miR5200 in wheat will be clarified, thereby providing more direct theoretical support for the precise application of tae-miR5200 in wheat stress tolerance improvement and molecular breeding.

## 5. Conclusions

This study identified 106 candidate target genes for tae-miR5200 through target gene prediction and validated the targeted interaction between tae-miR5200 and the photoperiod gene *VRN3* using a GUS reporter system. Building on this foundation, we performed preliminary screening via bioinformatics analysis. Combined with functional validation through tobacco transient transfection experiments, seven active functional loci capable of generating mature tae-miR5200 (tae-MIR5200-7A2/7A3/7A5/7B1/7B2/7B3/7D1) were identified from 13 potential tae-miR5200 gene loci. This identification effectively eliminates interference from pseudogene loci and provides precise functional research targets as well as a solid experimental foundation for systematically deciphering the complete functional chain of tae-miR5200. For the identified active tae-MIR5200 loci, *cis*-acting element analysis of their upstream promoter regions revealed enrichment of light-response, stress-response, and hormone-response elements. Combined with expression pattern analysis under different stress treatments, this further confirmed that the active loci of tae-miR5200 respond to drought, low-temperature stress, and hormone signals. In summary, this study not only identified the active gene loci of wheat tae-miR5200 but also provided crucial experimental evidence and theoretical foundations for further exploring the molecular mechanisms of tae-miR5200 in regulating wheat growth and development and adapting to environmental stress. This study provides the first experimental evidence for active tae-miR5200 loci with potential use in stress-tolerant wheat breeding. Additionally, these active loci open up a new pathway for the directed improvement in wheat stress tolerance and stable-yield breeding, and the technical framework established in this study can also serve as a reference paradigm for the excavation and functional analysis of miRNAs with unknown functions in other crops.

## Figures and Tables

**Table 1:** Minimum free energy of secondary structures for tae-miR5200 gene loci.

Gene Locus	Chromosomal Location	Free Energy (kcal/mol)	Gene Locus	Chromosomal Location	Free Energy (kcal/mol)
tae-MIR5200-2A	2A: 704714505–704714525 (-)	−17.1	tae-MIR5200-7B2	7B2: 9130407–9130427 (+)	−37.0
tae-MIR5200-7A1	7A1: 71680051–71680071 (-)	−27.7	tae-MIR5200-7B3	7B3: 9131147–9131167 (+)	−37.5
tae-MIR5200-7A2	7A2: 72697130–72697150 (-)	−38.1	tae-MIR5200-7D1	7D1: 67104771–67104791 (+)	−36.3
tae-MIR5200-7A3	7A3: 72698427–72698447 (-)	−37.7	tae-MIR5200-7D2	7D2: 67105364–67105384 (+)	−34.5
tae-MIR5200-7A4	7A4: 72699176–72699196 (-)	−30.3	tae-MIR5200-7D3	7D3: 68403842–68403862 (+)	−39.8
tae-MIR5200-7A5	7A5: 72700597–72700617 (-)	−36.6	tae-MIR5200-Un	Un: 84099233–84099253 (+)	−32.4
tae-MIR5200-7B1	7B1: 9129812–9129832 (+)	−35.3			

**Table 2:** Top 8 of predicted target genes of tae-miR5200.

Target Gene	Expectation	Alignment	Inhibition
TraesCS3A02G143100	2.5	:::::::::::::: .:.::	Cleavage
TraesCS3B02G162000	2.5	:::::::::::::: .:.::	Cleavage
TraesCS3D02G144500	2.5	:::::::::::::: .:.::	Cleavage
TraesCS7A02G115400	2.5	: ::::::::::::::: ::	Cleavage
TraesCS7B02G013100	2.5	: ::::::::::::::: ::	Cleavage
TraesCS7D02G111600	2.5	: ::::::::::::::: ::	Cleavage
TraesCS3B02G000400	2.5	::::::: ::::::::::	Translation
TraesCS3A02G001500	2.5	::::::: ::::::::::	Translation

**Table 3:** Comparison of information between functional and non-functional loci.

Gene Locus	Functional Activity (Yes/No)	Minimum Free Energy/(kcal/mol)	Secondary Structure
tae-MIR5200–7A2	Yes	−38.1	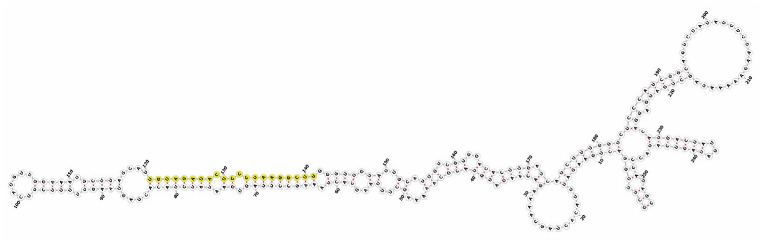
tae-MIR5200–7A3	Yes	−37.7	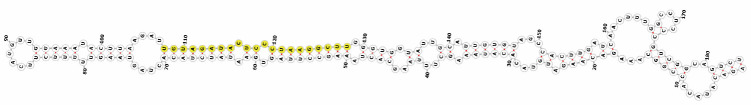
tae-MIR5200–7A5	Yes	−36.6	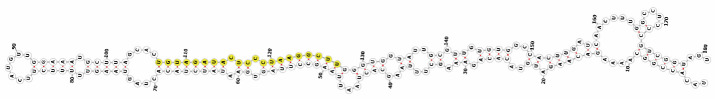
tae-MIR5200–7B1	Yes	−35.3	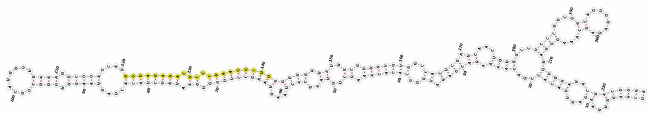
tae-MIR5200–7B2	Yes	−37.0	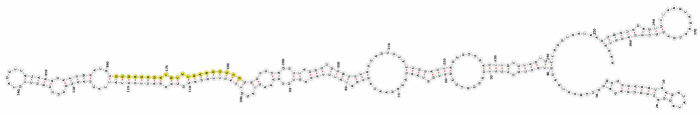
tae-MIR5200–7B3	Yes	−37.5	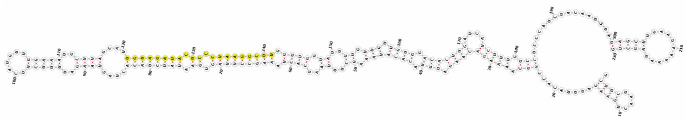
tae-MIR5200–7D1	Yes	−36.3	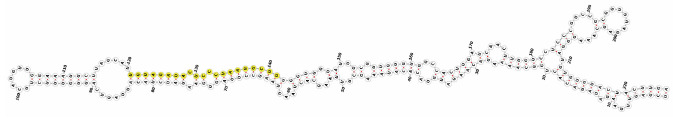
tae-MIR5200–2A	No	−17.1	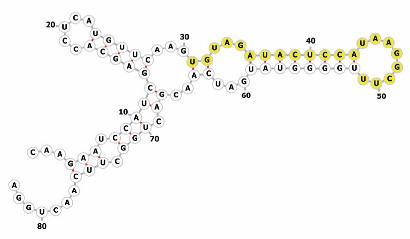
tae-MIR5200–7A1	No	−27.7	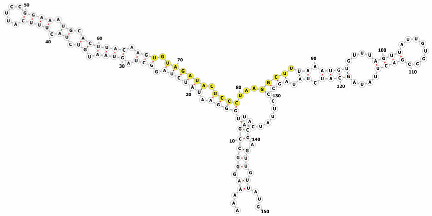
tae-MIR5200–7A4	No	−30.3	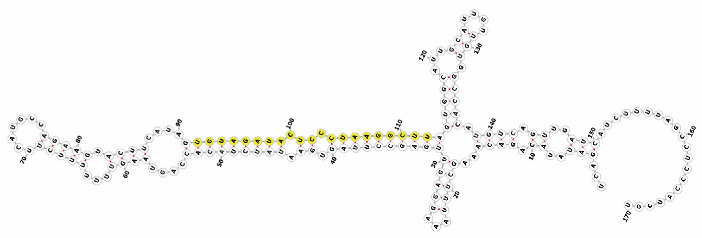
tae-MIR5200–7D2	No	−34.5	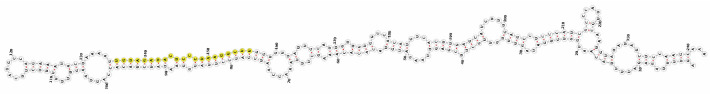
tae-MIR5200–7D3	No	−39.8	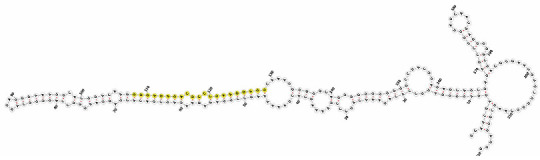
tae-MIR5200–Un	No	−32.4	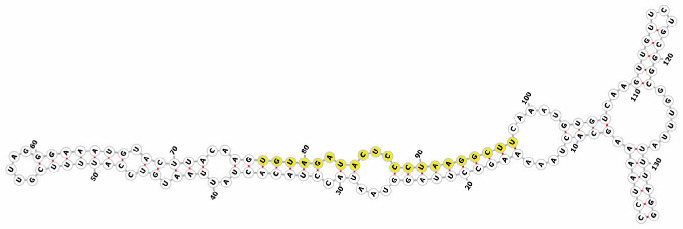

## Data Availability

The original contributions presented in this study are included in the article. Further inquiries can be directed to the corresponding author.

## Supplementary Materials

The following supporting information can be downloaded at: https://www.mdpi.com/article/10.3390/genes16111349/s1, Table S1: Primers for gene locus identification; Table S2: Primers for expression analysis; Table S3: Predicted target genes of tae-miR5200; Table S4: *Cis*-acting elements in the promoter region of the tae-miR5200-A2, tae-miR5200-A3, tae-miR5200-A5, tae-miR5200-B1, tae-miR5200-B2, tae-miR5200-B3, tae-miR5200-D1; Table S5: Numerical fold change of miR5200 relative expression level in stress responses; Text S1: The components of the nutrient solution; Text S2: Specific experimental procedures for vector construction; Text S3: Specific experimental procedures for agrobacterium-mediated tobacco transient transformation; Text S4: Specific experimental procedures for qRT-PCR; Figure S1: Schematic diagram of the experimental workflow.
