# Supplementary material for: Genome-Wide Identification of 13 miR5200 Loci in Wheat and Investigation of Their Regulatory Roles Under Stress
Source: Genes (Basel). 2025 Nov 9;16(11):1349. doi: 10.3390/genes16111349 (PMC12652891; doi:10.3390/genes16111349)
Supplement: Supplementary file 1 [file genes-16-01349-s001.zip › Text S1.pdf]

## Composition of 1 kg nutrient solution:

470 – 480 mg calcium nitrate tetrahydrate,
30 – 40 mg potassium dihydrogen phosphate,
130 – 140 mg potassium sulfate,
160 – 170 mg magnesium sulfate heptahydrate,
5 – 10 mg iron(III) chloride hexahydrate,
0.1 – 0.5 mg manganese(II) chloride tetrahydrate,
1 – 2 mg boric acid / 0.1 – 0.5 mg copper(II) sulfate pentahydrate,
0.5 – 1.0 mg zinc sulfate heptahydrate,
0.1 – 0.5 mg ammonium molybdate.
The solvent is distilled water.
