# Supplementary material for: Genome-Wide Identification of 13 miR5200 Loci in Wheat and Investigation of Their Regulatory Roles Under Stress
Source: Genes (Basel). 2025 Nov 9;16(11):1349. doi: 10.3390/genes16111349 (PMC12652891; doi:10.3390/genes16111349)
Supplement: Supplementary file 1 [file genes-16-01349-s001.zip › Text S2.pdf]

Specific experimental procedures for vector construction：

RNA extraction from Chinese Spring wheat was performed according to the TransZol kit protocol (TransGen Biotech, Beijing, China). RNA concentration was measured using the NanoDrop2000 (NanoDrop Technologies, Wilmington, DE, USA). cDNA synthesis was conducted following the Hifair III 1st Strand cDNA Synthesis Kit protocol (Yeasen Biotech, Shanghai, China). PCR amplification was performed using primers listed in Table S1. The SanPrep Column DNA Gel Extraction Kit (Sangon Biotech, Shanghai, China) was used to recover PCR products from the agarose gel. The recovered gene fragments were cloned into the pCAMBIA3301 plasmid vector using the Hieff Clone Plus One Step Cloning Kit (Yeasen Biotech, Shanghai, China). The vector was transformed into E. coli DH5α competent cells (Yeasen Biotech, Shanghai, China). A single-colony culture was sent to AuGCT Biotech (Wuhan, China) for sequencing, which confirmed the successful construction of the recombinant plasmid.
