# Supplementary material for: Genome-Wide Identification of 13 miR5200 Loci in Wheat and Investigation of Their Regulatory Roles Under Stress
Source: Genes (Basel). 2025 Nov 9;16(11):1349. doi: 10.3390/genes16111349 (PMC12652891; doi:10.3390/genes16111349)
Supplement: Supplementary file 1 [file genes-16-01349-s001.zip › Text S3.pdf]

Specific experimental procedures for agrobacterium-mediated tobacco transient transformation:

Agrobacterium tumefaciens EHA105 competent cells (Weidi Biotech, Shanghai, China) were transformed with the constructed recombinant plasmid. A single colony was selected for PCR identification, followed by inoculation into 10 mL YEB liquid medium supplemented with 50 mg/L kanamycin and 50 mg/L rifampicin. The inoculated medium was then cultured at 28℃ with shaking at 200 rpm for 16-20 hours. The bacterial pellet was collected by centrifugation at 5000 rpm for 3 minutes and resuspended in infiltration buffer formulated as follows: 50 mM MES; 2 mM Na_3PO_4 ; 0.5% (w/v) glucose; 150 μM acetosyringone. The concentration of the resuspended bacterial suspension was adjusted to approximately 0.8 OD_{600} for tae-MIR5200, and 0.6 OD_{600} for the empty vector (EK) and target gene (*VRN3*).
