# Supplementary material for: Genome-Wide Identification of 13 miR5200 Loci in Wheat and Investigation of Their Regulatory Roles Under Stress
Source: Genes (Basel). 2025 Nov 9;16(11):1349. doi: 10.3390/genes16111349 (PMC12652891; doi:10.3390/genes16111349)
Supplement: Supplementary file 1 [file genes-16-01349-s001.zip › Text S4.pdf]

Specific experimental procedures for qRT-PCR：

Extract RNA according to the TransZol® Kit (TransGen Biotech, Beijing, China) instructions. Depending on experimental requirements, synthesize conventional cDNA and cDNA for miRNA expression detection. For conventional cDNA synthesis, the reaction was performed using the Hifair® III 1st Strand cDNA Synthesis SuperMix for qPCR (gDNA digester plus), following the manufacturer’s instructions. qRT-PCR was performed using Hieff® qPCR SYBR® Green Master Mix (No Rox) (Yeasen Biotech, Shanghai, China), adhering to the manufacturer’s instructions specific to the CFX96 instrument (Bio-Rad, CA, USA).

PCR parameters were configured as follows: pre-denaturation was conducted at 95℃ for 5 min first, then 40 cycles were run. Each cycle consisted of 10 s of denaturation at 95℃, 30 s of annealing at 60℃, and 20 s of extension at 72℃.
