# Supplementary figures and images for: Genome-Wide Identification of 13 miR5200 Loci in Wheat and Investigation of Their Regulatory Roles Under Stress
Source: Genes (Basel). 2025 Nov 9;16(11):1349. doi: 10.3390/genes16111349 (PMC12652891; doi:10.3390/genes16111349)

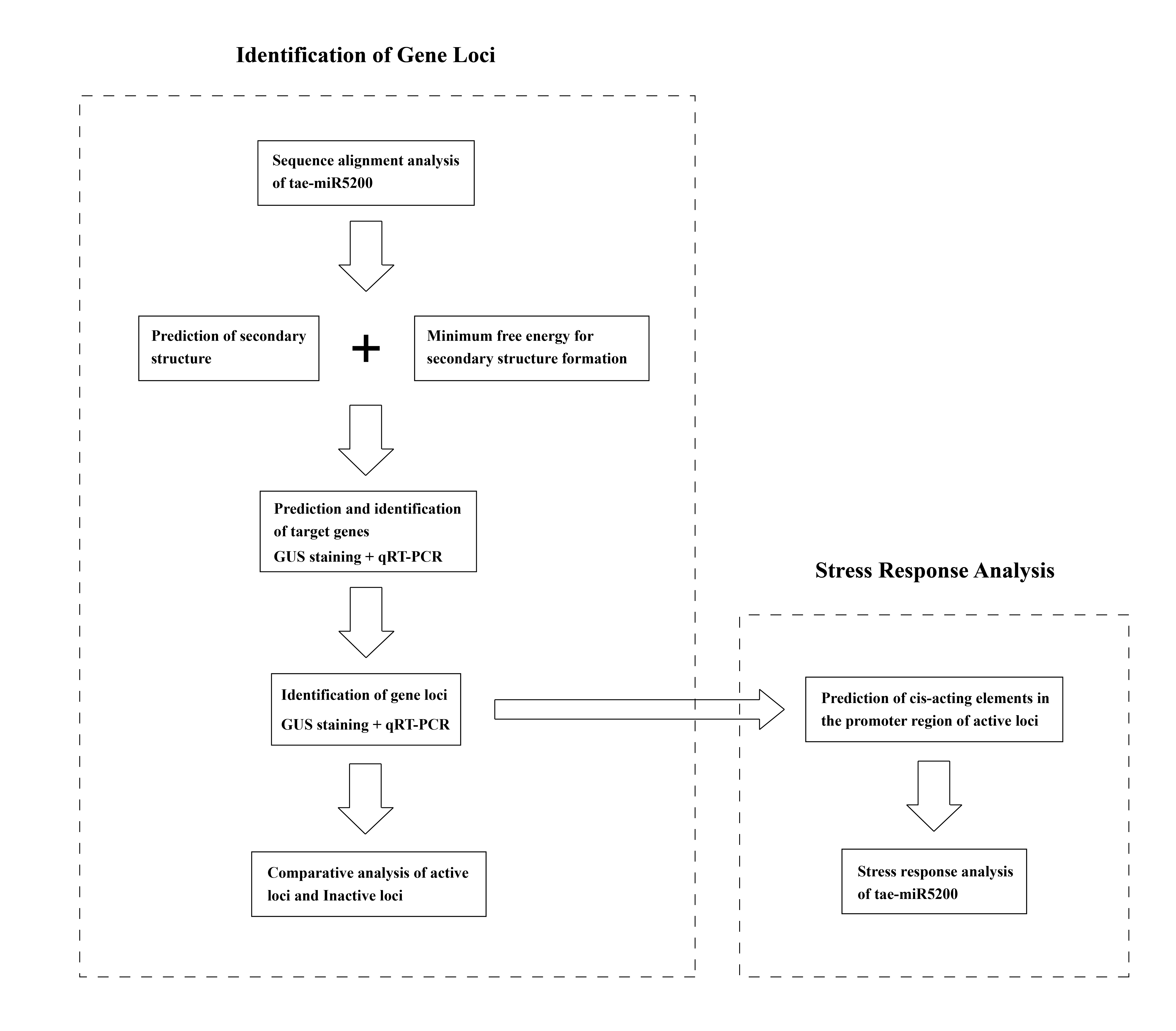

Supplement: Supplementary file 1 [file genes-16-01349-s001.zip › Figure S1.jpg]
